# Neighborhood Greenery as a Predictor of Outdoor Crimes between Low and High Income Neighborhoods

**DOI:** 10.3390/ijerph17051470

**Published:** 2020-02-25

**Authors:** Young-Jae Kim, Eun Jung Kim

**Affiliations:** 1 Department of Forest Resources and Landscape Architecture, Yeungnam University, Gyeongbuk 38541, Korea; youngjae_kim@yu.ac.kr; 2 Department of Urban Planning, Keimyung University, Daegu 42601, Korea

**Keywords:** neighborhood safety, outdoor crimes, neighborhood greenery, green vegetation, low and high income

## Abstract

Neighborhood greenery contributes to improving mental, emotional, and physical health and may help to promote neighborhood safety. Several studies have reported positive effects of neighborhood greenery on the improvement of outdoor safety, but little is known about whether the relationship between green vegetation and outdoor safety varies with the income status of neighborhoods. The purpose of this study is to examine neighborhood greenery as a predictor of outdoor crime rates between low and high-income neighborhoods while controlling for the sociodemographic conditions of the neighborhoods. This study used 2010 census block group data and objectively measured natural environment data derived from GIS in Austin, Texas. Comparison t-tests and ordinal least square regressions were conducted as statistical analyses. The t-tests showed that low-income neighborhoods were more socioeconomically disadvantaged and had less greenery than high-income neighborhoods. The final regression models showed that neighborhood greenery had a negative relationship with outdoor crimes for low-income neighborhoods but a positive relationship with crimes for high-income neighborhoods. The results suggest that different strategies may be needed in dealing with neighborhood safety according to neighborhood-level income.

## 1. Introduction

A growing body of studies has shown that living in green areas has promoted health. Ulrich et al. (1991) found that exposure to the natural environment, including nature vegetation and water, could affect stress recovery [1]. Since then, many studies indicated that the greenness of a community is one of the most important factors in health outcomes (e.g., physical activity, mental health, disease) [1,2,3,4,5,6,7], environmental conditions (e.g., air quality, noise) [8,9,10,11], and social cohesion [7,11,12,13,14].

Looking at the benefits of greenness of a community, many studies have focused on the health-enhancing effects of the green spaces [2,3,4,5,6,7]. Especially, James et al. (2015) systematically reviewed 66 studies that had been conducted until recently to identify the health benefits of exposure to greenness. The study designs were cross-sectional, prospective, birth cohort, experimental, and ecological. They found that higher greenness was correlated to physical activity (+), overweight or obesity (−), self-reported mental health (+), birth weight (+), cardiovascular disease (−), and mortality (−) [2].

Greenness has other benefits, such as reducing air pollution and noise and promoting social interaction. As the environmental benefits of green spaces, Nowak et al. (2006) investigated the effect of urban trees on removal of pollution (O_3_, CO, NO_2_, PM_10_, and SO_2_) for the entire of United States using computer modelling. They found that urban trees had significant effects on reducing air pollution [8]. Gidlöf-Gunnarsson and Öhrström (2007) were interested in the noise-reducing effects of the green environment, which they conducted a cross-sectional questionnaire study in Stockholm and Göteborg, Sweden. They found that people living near the green areas were less likely to suffer from noise and psychological stress [9]. In terms of mitigating the thermal stress of green spaces, Lafortezza et al. (2009) proved that the visit of green spaces in the period of heat stress was effective in reducing the perception of thermal discomfort [10]. Meanwhile, Markevych et al. (2017) proposed pathways linking green areas to health outcomes. They found that green space reduces exposure to air pollution and noise and affects overall health. They also found that greener areas could also promote attention restoration, reduce stress, encourage physical activity, and facilitate social interaction [11]. Likewise, some studies have demonstrated that green environment improved social interactions as well [7,12,13,14]. Recently, the importance of greenness in the urban context has emerged in promoting the safety of neighborhood environments [15,16,17,18]. Branas et al. (2015) examined the impact of a greening program for vacant lots on the safety and health in Philadelphia, Pennsylvania, in 1999 and 2008, using a quasi-experimental difference-in-differences study. They compared the lots treated with greening to control lots with no greening and found that greening was significantly associated with reduced gun assaults, vandalism, and stress while promoting exercise [15]. 

Kuo and Sullivan (2011) examined the relationship between vegetation and crime. They studied 98 apartment buildings in one inner-city neighborhood using police data. The results showed that vegetation density around buildings had a significantly negative correlation with the number of police crime reports [16]. Kim (2019) examined the relationship between urban green areas and outdoor crime rates in Austin, TX, and found that the Normalized Difference Vegetation Index (NDVI) and the park rates in the neighborhood were negatively correlated to the crime rate [18]. Lovasi et al. (2013) investigated the environmental correlates of safety and greenness in terms of the obesity rate among children in New York. They also found that a higher obesity rate was associated with a higher rate of homicide and lower density of street trees [17].

Some studies have used objectively measured indicators of safety. As objectively measured indicators of safety hazards, Branas et al. (2015) used data on crimes and arrests (e.g., aggravated assaults, robberies, burglaries, thefts, etc.) [15], and Lovasi et al. (2013) used the homicide rate and pedestrian-auto fatality rate [17]. However, many studies still use survey-based protocols to identify the levels of neighborhood safety [19,20,21,22,23,24,25]. To study environmental safety more accurately, it is important to measure the level of environmental safety objectively. Data for objectively measured crime incidents in outdoor neighborhoods as a safety indicator could be useful to advance our understanding of the association between the levels of greenness and safety.

Despite the importance of green spaces in promoting safety, green space in urban areas is disproportionately distributed. Some studies examined the regional inequality in access to green spaces according to socio-economic status. Most of these studies have shown that green spaces were less for socially and economically disadvantaged and ethnical minority neighborhoods [2,26,27,28,29,30]. In one such example, Duncan et al. (2013) examined the relationships between racial composition, poverty, and access to recreational open spaces in Boston, Massachusetts. Using a regression model, they found that there was a negative association between the percentage of non-Hispanic black people in census data and the density of open spaces [26]. Another study of an Australian case also found that a lower level of green space was related to lower levels of socio-economic status in neighborhoods [27]. On the other hand, some studies found that there were more and/or higher quality of green areas in low-income and minority areas [31,32]. For example, Engelberg et al. (2016) conducted a study in Seattle, WA and Baltimore, MD to examine socioeconomic and ethnic disparities in park quality. As a partial finding of the study found that low-income neighborhoods in Seattle, WA had better park quality on average [31].

Although some research has been conducted, there is still an insufficient link between green spaces in urban environments and the income levels of neighborhoods. More studies are needed to investigate the association between greenness, outdoor safety, and the level of income using objectively measured variables. Therefore, this study examines the differences in access to urban green areas according to neighborhood income levels. It also explores the impact of urban green spaces on neighborhood safety in areas with high and low levels of income using objectively measured variables.

## 2. Methods 

### 2.1. Study Area and Samples

The study area was Austin, the capital city of the US state of Texas. It is one of the fastest-growing large cities in the US and has a population of 964,254 as of 2019, which has increased by about 22% from 790,491 according to the 2010 census [33]. The violent crime rate is 3.91 per one thousand residents, which is higher than the rate for all communities of all sizes in the US but lower than that of comparably sized cities [34]. This means that Austin is actually safer than other communities of similar population size according to NeighborhoodScout’s exclusive analysis of FBI crime data [34]. Many crimes in Austin are property crimes with a rate of 36 per one thousand population. Thus, this study could contribute to the literature on the possibility of a new role of green space in promoting neighborhood safety for cities in the US with similar population size.

**Figure 1:**
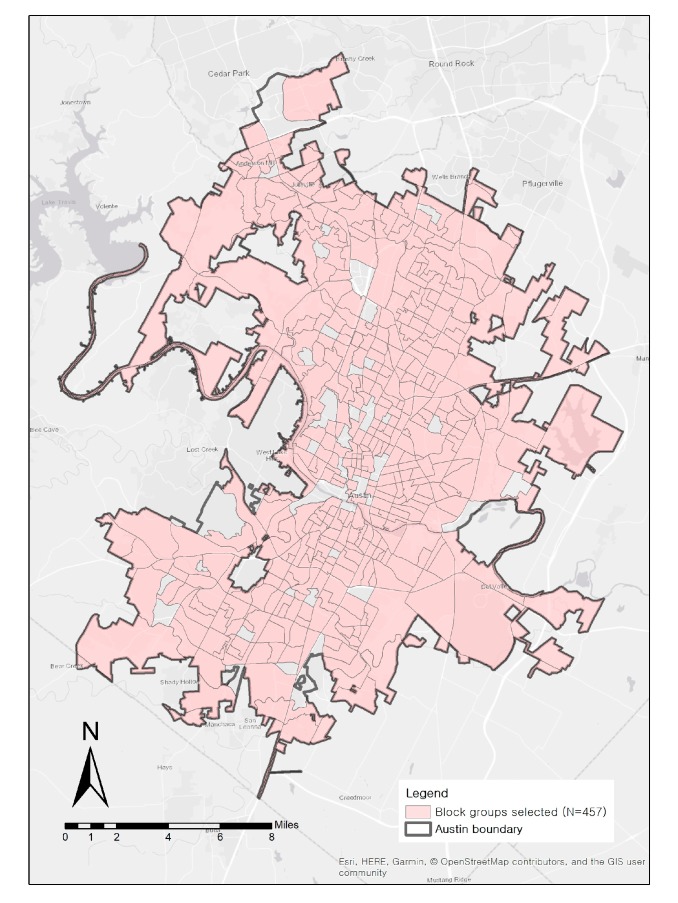
Study area and block groups (unit of analysis, *n* = 457).

Census block groups were used as a spatial unit of analysis for this study. Austin has a total of 506 block groups, and this study used 457 block groups after excluding 49 samples. The excluded block groups had median incomes less than $10,000, suggesting that a small number of residents live in the communities or that the block groups are heavily commercial areas. Figure 1 shows the study area and 457 block groups used in this study.

### 2.2. Measures

#### 2.2.1. Sociodemographic and Population (Confounding Variables) 

This study used 2010 census data to capture various sociodemographic conditions of neighborhoods. Seven sociodemographic variables were used to identify the levels of economically disadvantaged neighborhoods: minorities, income, median home prices, unemployment, female-headed families, and teenage school dropout [35,36,37,38,39,40]. The population density was also used (the number of residents per acre). These sociodemographic variables and the population density variable were used as confounding factors in statistical analyses.

#### 2.2.2. Neighborhood Greenery and Natural Environmental Conditions (Independent and Additional Confounding Variables)

The neighborhood greenery variables such as tree canopy, NDVI, parks, and water features were used as the main independent variables in this study. They were all indicators that directly represent the greenness of a community. Additionally, the natural environmental conditions including slope and surface temperature, which are indirectly related to the neighborhood greenery, were considered in this study as confounding factors. 

As many studies have shown earlier, green spaces have the effect of reducing crime rates [15,16,17,18]. However, some studies suggested that green spaces might rather play a role in increasing crime [41,42]. This is explained by the “eyes on the street” [43], which means that visibility is insufficient in green areas. In general, green areas in the neighborhoods are often terrain such as mountains and hills. In other words, it is difficult to be visible in areas such as mountains and hills with steep slopes because they are surrounded by dense forests. Therefore, this study included slope variable, in spite of no direct association between slope and crime rates. 

Meanwhile, some studies have supported that there was a positive linear relationship between temperature and violence [44,45,46,47]. However, some other studies insisted in an inverted-U pattern between them [48,49,50]. The reason for this curvilinear relationship was that people interacted less on very hot days, which in turn might result in a reduction in violent crimes. For example, in the case of curvilinear relationship, Gamble and Hess (2012) found that there was an inverted-U pattern with a threshold at about 90 °F between daily temperature and crimes including aggravated assaults, homicide, and rape in Dallas, Texas [50]. In any case, there was a correlation between temperature and crime in several studies, so this study employed a surface temperature variable. 

Data for tree canopy were obtained from the city of Austin. Each variable shows the percentage of tree canopy areas within block groups, respectively. The density of parks and water features available in neighborhoods were also captured for each block group. 

The NDVI was calculated for each pixel (30 × 30 m) for the whole area in Austin in geographic information systems (GIS). This was done using a remotely sensed image produced by the Landsat 5 Thematic Mapper (TM) on 4 August 2009. The image did not have any cloud obstruction. The calculated values of NDVI range from –1 to 1, with values closer to 1 indicating greater dense green vegetation [51]. The captured NDVI was the mean of NDVI for each block group. The surface temperature was also derived from the Landsat 5 TM image as the average of air temperature for each block group. Steep slopes greater than 5% and 8.33% were measured using a digital elevation modeling process in GIS. The slope variables indicate the percentage of steep slopes greater than 5% and 8.33% at the block group level. The timeline for the original data acquisition of the greenery and other natural environmental conditions was between 2009 and 2011, which matches with the time for the 2010 census data.

#### 2.2.3. Neighborhood Safety (Dependent Variable) 

Neighborhood safety was captured from crime point data from 2005 to 2009, which were obtained from the Austin Police Department. The crime data specify the locations and types of crimes in Austin. In contrast to other crime data (all types of crimes) objectively measured to capture neighborhood safety [25], two particular types of crimes, kidnapping and sexual assault, were used for statistical analyses because they are more likely to occur in outdoor neighborhoods and may account for neighborhood safety [18]. Especially, fear of sexual assault in neighborhoods has been documented as factors constraining physical activity and outdoor recreation in several previous studies [52,53,54]. Furthermore, parental concerns about neighborhood safety, particularly the dangers associated with strangers or stranger abductions, have also been identified as one of the main deterrents to children’s active transport [55,56]. Out of the total number of 1,010,773 crimes during the past five years, 3871 crime incidents were used for the final sample. After geocoding the spatial locations of the crime incidents in GIS, the yearly outdoor crime rates per acre were generated for the neighborhood safety variable, which were calculated from total number of crimes during the five years divided by block group areas in acres and divided by 5. 

### 2.3. Data Analysis

A t-test was conducted to compare the mean of the study variables between low and high-income neighborhoods. The null hypothesis for the t-tests was that there are no differences in the mean sociodemographic characteristics and natural environmental conditions (neighborhood greenery) between low and high-income neighborhoods. Block groups were categorized into low income and high-income neighborhoods based on whether the median income of a block group was greater than $50,000 [57]. The block groups with a median income less than $50,000 were coded as zero, representing low-income neighborhoods, and the rest were coded as one, representing high-income neighborhoods. 

An ordinary least square (OLS) regression model was used with the outcome variable of neighborhood safety captured by a continuous scheme (yearly outdoor crime rates). First, a series of one-by-one tests were conducted by adding each natural environment variable to the base model one at a time, which included all the confounding variables. The one-by-one tests examine the relationship between one natural environmental variable and neighborhood safety while controlling for sociodemographic factors. Second, a final model including the confounding factors and all significant variables of the natural environment was generated for each low and high-income neighborhood. All of the statistical analyses were done using STATA version 14 [58].

## 3. Results 

### 3.1. Bivariate Tests

#### 3.1.1. Sociodemographic Characteristics

Table 1 shows the results of the differences in the mean of sociodemographic characteristics and population density between low- and high-income neighborhoods. All the sample characteristics of block groups were significantly different between low- and high-income neighborhoods. The percentage of minorities in low-income neighborhoods was 54.65%, which is almost two times higher than in high-income neighborhoods, meaning that more than half of residents living in low-income neighborhoods are minorities. The mean household incomes in low and high-income neighborhoods were $33,367 and $75,084, respectively. The home price was $88,380 higher in high-income neighborhoods. Low-income neighborhoods had higher unemployment (5.62% vs. 2.53%), more female-headed families (27.78% vs. 14.41%), more teenage school dropouts (22.91% vs. 5.14%), and received more welfare (2.53% vs. 0.81%). The population density per acre was four times higher for low-income than high-income neighborhoods, but the difference was not statistically significant at the level of 0.05.

#### 3.1.2. Neighborhood Greenery

Table 2 presents the mean differences in the natural environmental characteristics between low income and high-income neighborhoods. The amount of tree canopy area was 10.17% higher for high-income neighborhoods, while surface temperature was 0.86 °C higher in low-income neighborhoods. The levels of neighborhood greenery measured by NDVI were higher for high-income neighborhoods. High-income neighborhoods also had more water features and more steep slopes.

### 3.2. Multivariable Analyses

Table 3 presents the results from the OLS regressions estimating the natural environmental correlates of neighborhood safety for each low income and high-income neighborhood while controlling for the sociodemographic variables. The numbers of minorities were positively associated with outdoor crime rates for both low and high-income neighborhoods. However, higher household income was associated with lower outdoor crime rates for low-income and high-income neighborhoods. Outdoor crime rates were positively associated with the percentage of teenage school dropouts increased, but the relationship was significant for only low-income neighborhoods. Higher rates of unemployment and welfare were positively associated with outdoor crime rates for high-income neighborhoods only. In the one-by-one models, surface temperature appeared to be positively associated with outdoor crime rates for low-income neighborhoods only. However, the tree canopy variable showed opposite relationships with outdoor crime rates according to the income levels, indicating that more tree canopies were associated with decreased outdoor crime rates for low-income neighborhoods but increased rates for high-income neighborhoods.

For the results from the final models for each-income neighborhood, two variables were not used due to multi-collinearity among the natural variables: tree canopy and surface temperature. For example, neighborhoods with higher levels of neighborhood greenery measured by NDVI were more likely to have a greater amount of tree canopy and lower surface temperature, as expected. Thus, one variable among these three variables should be used to develop the final model. The NDVI variable showed greater coefficients in the one-by-one models (−2.943 for low-income neighborhoods and 0.478 for high-income neighborhoods), so it was selected. 

As predicted by the tree canopy variable in the one-by-one models, when the NDVI variable used as an indicator of neighborhood greenery, there were different relationships with outdoor crime rates according to income levels. Higher NDVI and more parks were negatively associated with outdoor crime rates for low-income neighborhoods but positively associated with the rates for high-income neighborhoods. The percentage of steep slopes greater than 8.33% showed a positive relationship with outdoor crime rates for low-income neighborhoods. 

**Figure 2:**
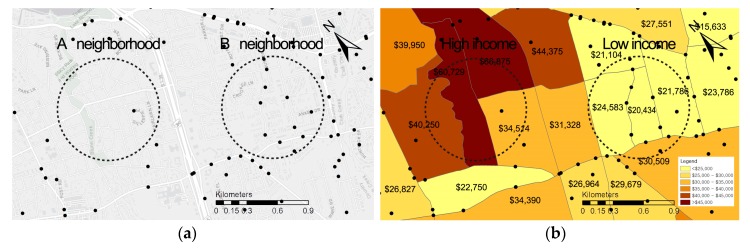

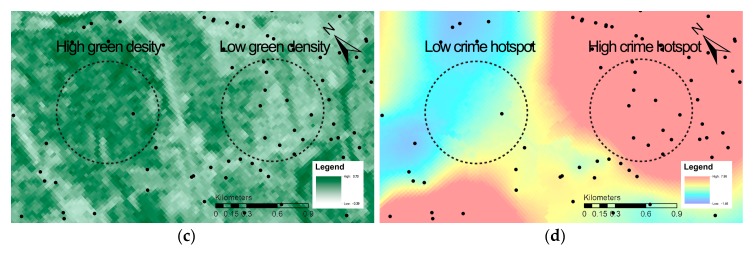
Comparisons of neighborhood characteristics between neighborhoods A and B: (**a**) base map; (**b**) income; (**c**) green density; (**d**) crime hotspots (neighborhood safety).

Crime hotspots were analyzed in terms of greenery and income levels between two selected neighborhoods, A and B. The dashed circles representing neighborhoods A and B in Figure 2 have radiuses of 500 meters. These neighborhoods have different levels of median income, environmental conditions in terms of green density, and crime hotspots. Neighborhood A has relatively high income, higher green density, and lower crime hotspots, whereas Neighborhood B has low income, lower green density, and higher crime hotspots.

## 4. Discussion

The findings from the bivariate tests revealed that the sociodemographic characteristics and the levels of neighborhood greenery vary with the income status of neighborhoods. Low-income neighborhoods were generally more socioeconomically disadvantaged than high-income neighborhoods. Low-income neighborhoods also had greater numbers of minorities, home prices, unemployment, female-headed families, teenage school dropout rates, and people receiving welfare than the high-income neighborhoods. This finding is pretty obvious because the low-income neighborhoods were defined based on income levels less than $50,000, and the odds of being socioeconomically disadvantaged increased. 

There were interesting findings about the different levels of neighborhood greenery between the low and high-income neighborhoods. More tree canopies, green vegetation measured by NDVI, and parks were observed for high-income neighborhoods. In contrast, higher surface temperatures were observed in low-income neighborhoods, which were associated with less coverage by green vegetation. This finding supports results that showed a positive correlation between levels of income and access to green space in neighborhoods [26,27]. 

The results from the multivariate regressions demonstrated that neighborhood safety varies with sociodemographic characteristics for both low income and high-income neighborhoods. Neighborhood safety appeared to increase if there were fewer minorities and when household income increased for both low and high-income neighborhoods. However, the teenage school dropout rate was associated with decreased neighborhood safety for low-income neighborhoods only, while increased rates of unemployment and those receiving welfare were associated with decreased neighborhood safety for high-income neighborhoods. These varying relationships might indicate that negative impacts of socioeconomic factors such as unemployment and welfare on neighborhood safety become more significant for high-income neighborhoods [59]. 

The results from the OLS regression models estimating the associations of neighborhood greenery with outdoor safety demonstrated that the roles of green vegetation in neighborhoods in impacting outdoor crime rates vary with the income status of neighborhoods. Increases in green vegetation in neighborhoods measured by NDVI were associated with increased neighborhood safety for low-income neighborhoods but with decreased neighborhood safety for high-income neighborhoods. This finding is consistent with several previous studies conducted by Kuo and Sullivan [16]. They examined samples of residents living in areas within a large public housing development in Chicago, who were mostly African American, unemployed, and received federal assistance. They showed negative relationships between the density of green vegetation (trees and grass) around buildings and the number of crimes per building reported [16]. One possible reason for the relationship between vegetation and outdoor crimes in high-income neighborhoods might be related to denser trees hindering visibility [60]. High-income neighborhoods are generally designated by low density with a small number of street intersections and a small number of residents in an area. Therefore, residents are more likely to use a vehicle instead of walking or bicycling in neighborhoods. 

In addition to NDVI, another measure of green vegetation, tree canopies, presented the same relationship direction for both low and high-income neighborhoods in the one-by-one models. The positive relationship of parks with neighborhood safety remained significant for only low-income neighborhoods. Furthermore, in the one-by-one models, additional increases in surface temperature were negatively associated with neighborhood safety for low-income neighborhoods. This finding may imply that crime hotspot areas generally have less green areas and thus have high impervious surface cover and high air temperatures [15,16,18,61]. This was verified by the results of a visual comparison analysis of two different neighborhood characteristics conducted in this study. Regarding the relationship between water features and outdoor crimes, this study did not show any significant results in one-by-one models and the final models for both low- and high-income neighborhoods. The reason this study used the water feature variable as one of the independent variables was because blue spaces (e.g., river, ponds, etc.) are resting places for residents along with green spaces in the community, which was expected to lead to many activities such as walking or bicycling in green and blue areas [62]. A future study may want to expand the research scope by examining whether blue spaces (water features) have the effects of natural surveillance on “eyes on the street”, which would have a positive effect on crime prevention in the community.

Furthermore, steep slopes appeared to be negatively associated with safety for low-income neighborhoods. This might be because visibility is insufficient in areas with steep slopes. Hilly terrain may also not be a desirable environmental condition for residents with low income status due to greater physical effort needed. Although people with low income are more likely to walk in their neighborhoods instead of driving due to no car ownership, several studies found that steep slopes are a factor that discourages people from engaging in walking or bicycling in neighborhoods [63,64].

This study has several limitations. First, for the measure of neighborhood safety, only two types of crimes were used: kidnapping and sexual assault. The assumption for using those two crimes as an indicator of neighborhood safety was based on the findings of previous studies, which indicated that fear of those crimes is very much an issue in terms of engaging in outdoor recreation and physical activity [52,53,54,55,56]. However, in general, fear of crimes and the reality of crimes are quite separate issues, and whether these crimes are really less likely to occur in outdoor neighborhoods, particularly in overgrown or heavily vegetated green space, should be identified in future studies. Our comparison analysis of neighborhoods A and B, shown in Figure 2, represented the possibility that higher greenery levels is associated with less crime, and the results from the OLS regressions supported the relationship for low income neighborhoods. To better understand the effects of neighborhood greenery on outdoor crimes, future studies need to consider more specific information about crime locations when it comes to determining neighborhood safety. Second, this study used census block groups as a unit of analysis, which might have been a limitation because all the data were clustered, and no richer sociodemographic characteristics or other natural environmental conditions were attained. Thus, future studies could use survey protocols to feature sample characteristics and natural environmental conditions based on the geocoded homes in ArcGIS. Third, this study categorized neighborhood groups based on a household income of $50,000. Considering a different cutoff point for income levels may provide somewhat different study results. However, the neighborhoods were categorized based on the literature and had comparable numbers of samples for both neighborhoods. Lastly, since this study was a cross-sectional design, future studies need to consider the causal relationships between study variables and to explore detailed mechanisms underlying these relationships. Moreover, a longitudinal research design is required to examine the effect of changes in neighborhood greenery levels on outdoor crime rates as a future study.

## 5. Conclusions

The role of neighborhood greenery in outdoor crime rates varies with the income status of neighborhoods. More green vegetation such as trees, grass, and parks can promote neighborhood safety for low-income neighborhoods, but it may negatively impact high-income neighborhoods. Providing more green space to help mitigate land surface temperature and providing walking-friendly built environments such as gentle slopes could help reduce outdoor crimes for low-income neighborhoods. These measures might create more visibility and promote neighborhood safety. For high-income neighborhoods, dealing with personal factors or socio-economic conditions may be effective to improve neighborhood safety, as shown by the results in this study. Future research examining the relationships between neighborhood greenery and outdoor crimes should be conducted in various settings with different sizes of cities, as well as different testing methods, such as experimental methods, survey protocols, and other census data.

## Figures and Tables

**Table 1:** Sample characteristics of low- and high-income neighborhoods and results of bivariate tests.

Sample Characteristics	Low Income (*N* = 282)	High Income (*N* = 213)	Difference in Mean
	Mean (SD)	Mean (SD)	
Minority (%)	54.65 (1.60)	23.49 (1.10)	31.16 ***
Household Income ($)	33,367 (9815)	75,084 (24,540)	−41,717 ***
Housing Value ($)	101,179 (62,141)	189,559 (109,587)	−88,380 ***
Unemployment (%)	5.62 (4.60)	2.53 (2.02)	3.09 ***
Female-Headed Family (%)	27.78 (19.33)	14.41 (12.40)	13.37 ***
Teenage School Dropout (%)	22.91 (23.17)	5.14 (10.14)	17.76 ***
Welfare Receipt (%)	2.53 (3.35)	0.81 (1.42)	1.72 ***
Population Density (num. per acre)	240.64 (3830)	55.12 (326)	185.52

*** *p* < 0.01.

**Table 2:** Neighborhood greenery in low- and high-income neighborhoods and bivariate test results.

Sample Characteristics	Low Income (*N* = 282)	High Income (*N* = 213)	Difference in Mean
	Freq. (%)	Freq. (%)	
Tree canopy (%)	29.27 (12.22)	39.44 (15.80)	−10.17 ***
NDVI (ranging from −1 to 1)	0.21 (0.07)	0.28 (0.09)	−0.07 ***
Park (%)	6.89 (12.80)	8.36 (13.90)	−1.47
Water features (%)	1.21 (4.07)	2.60 (10.67)	−1.39 **
Surface temperature (°C)	32.54 (2.20)	31.67 (1.65)	0.86 ***
Steep slope >5% (%)	11.47 (9.45)	14.47 (11.93)	−3.00 ***
Steep slope >8.33% (%)	5.51 (6.16)	8.65 (10.43)	−3.14 ***

** 0.01 ≤ *p* < 0.05; *** *p* < 0.01.

**Table 3:** Ordinary least square (OLS) regression models estimating the associations of neighborhood greenery with outdoor crime rates for low and high-income areas.

Sample Characteristics	Low Income				High Income			
	One-by-One Models		Final Model		One-by-One Models		Final Model	
	Coef.	P > |t|	Coef.	P > |t|	Coef.	P > |t|	Coef.	P > |t|
**Socio-demographic**								
Minority (%)			0.010	0.029			0.011	<0.000
Income ($)			−0.020	0.056			−0.002	0.044
Housing Value ($)			−0.002	0.327			0.000	0.249
Unemployment (%)			−0.013	0.573			0.019	0.053
Female-Headed Family (%)			0.002	0.635			0.001	0.465
Teenage School Dropout (%)			0.011	0.004			−0.002	0.267
Welfare Receipt (%)			−0.017	0.548			0.045	0.001
Population Density (num. per acre)			0.002	0.448			0.000	0.411
**Natural environment**								
Tree canopy (%) ^†^	−0.014	0.063			0.002	0.069		
NDVI (ranging from −1 to 1) ^†^	−2.943	0.019	−2.589	0.047	0.478	0.041	0.572	0.023
Park (%) ^†^	−0.015	0.024	−0.013	0.065	−0.001	0.345	−0.001	0.596
Water features (%) ^†^	−0.024	0.238			−0.002	0.146		
Surface temperature (°C) ^‡^	0.388	<0.000			0.008	0.585		
Steep slope >5% (%) ^‡^	0.007	0.438			0.001	0.534		
Steep slope >8.33% (%) ^‡^	0.016	0.264	0.024	0.085	0.000	0.957	−0.002	0.219
**Number of Observations**				256				201
**Adjusted R-squared**				0.1902				0.4496
**Root MSE**				1.2673				0.2462

Note: ^†^ These variables indicate the neighborhood greenery and were treated as independent variables. ^‡^ These variables were used as confounding factors for the final regression models.
